# Body Composition Changes and Their Associations with Physical Activity and Screen Time in a Sample of Italian Early Adolescents over a 3-Year Period

**DOI:** 10.3390/children13010130

**Published:** 2026-01-15

**Authors:** Emanuela Gualdi-Russo, Stefania Toselli, Federica De Luca, Gianni Mazzoni, Simona Mandini, Sabrina Masotti, Luciana Zaccagni

**Affiliations:** 1 Department of Neuroscience and Rehabilitation, Faculty of Medicine, Pharmacy and Prevention, University of Ferrara, 44121 Ferrara, Italy; federica.deluca@unife.it (F.D.L.); gianni.mazzoni@unife.it (G.M.); simona.mandini@unife.it (S.M.); luciana.zaccagni@unife.it (L.Z.); 2 Department for Life Quality Studies, University of Bologna, 47921 Rimini, Italy; stefania.toselli@unibo.it; 3 Center for Exercise Science and Sports, University of Ferrara, 44123 Ferrara, Italy

**Keywords:** screen time, sport, anthropometry, growth, increments

## Abstract

Background: A sedentary lifestyle contributes to chronic disease risk in adults and may predict unfavourable body composition in adolescents. Declining physical activity and rising sedentary behaviour are linked to increasing global obesity rates. Given the scarcity of longitudinal studies examining how participation in organized sports and screen device use relate to body composition in early adolescence, this study aims to address this gap by analyzing temporal trends in both sexes. Methods: A sample of 158 Italian students, 38% of whom were female, was followed longitudinally from ages 11 to 13. Annual anthropometric assessments were conducted, and self-reported data on screen time and organised sports participation were collected. Fat mass (FM), fat-free mass (FFM), fat mass index (FMI), fat-free mass index (FFMI), body mass index (BMI), and waist-to-height ratio (WHtR) were subsequently calculated, along with annual increments. Repeated-measures ANOVA assessed age and sex effects, while multiple regression models evaluated associations between behavioural variables or sex and body composition indices. Results: Significant differences in %F, FM, FFM and its increment, WHtR and its increment, FMI, and FFMI (all *p* < 0.01) were observed by age and sex interaction. At age 13, weekly sports participation was negatively associated with annual increments in %F (*β* = −0.204, *p* = 0.04) and FMI (*β* = −0.227, *p* = 0.03). Female sex was associated with greater increments in %F (*β* = 0.188, *p* < 0.05) and WHtR (*β* = 0.323, *p* < 0.01), and with smaller increments in FFM (*β* = −0.421, *p* < 0.01). No significant associations were found for screen time (*p* > 0.05). Conclusions: Sporting during early adolescence seems to have positive effects on body composition changes, while sex-specific patterns warrant further attention. A deeper understanding of how early adolescent lifestyle factors, such as physical activity and sedentary behaviour, shape body composition is essential for promoting long-term health.

## 1. Introduction

Adolescence, spanning from ages 10 to 19, represents a crucial phase of growth and development during the transition from childhood to adulthood. Poor health conditions during this period can persist in adulthood [1]. Alongside the profound biological changes that characterize adolescence—varying in intensity, speed, and duration according to sex and genetic factors [2]—lifestyle-related exogenous factors also play an important role. Sports participation, in particular, can shape body composition and support mental well-being by fostering awareness and acceptance of physiological changes [3,4,5].

Obesity during adolescence is clinically relevant because it frequently tracks into adulthood: approximately 80% of adolescents with obesity remain obese as adults [6]. Globally, the prevalence of obesity among children and adolescents aged 5–19 years has risen substantially, from just 2% in 1990 to 8% in 2022 [7]. According to the 2022 Health Behaviour in School-Aged Children (HBSC) survey in Italy, 18.2% of adolescents aged 11–17 years were classified as overweight and an additional 4.4% as obese [8]. Consequently, the increasing prevalence of overweight and obesity among young people represents a major public health concern. It is largely linked to insufficient physical activity (PA) and sedentary behavior associated with intensive screen use [9,10]. Sedentary behavior—characterized by low energy expenditure in sitting or lying positions across daily contexts—has been consistently associated with poor body composition, excess weight, and metabolic syndrome [11,12]. Since overweight adolescents are more likely to become obese adults and face a higher risk of cardiovascular disease [13,14], the global increase in obesity among adolescents is prompting calls for stronger weight management interventions [15]. PA provides well-recognized physical, cognitive, and psychosocial benefits [16,17,18]. Higher PA levels and motor competence are associated with healthier weight status, whereas lower levels increase obesity risk [19].

Educational programs promoting healthy nutrition and exercise are effective, and organized physical activities show particular benefits [10,20,21]. Yet, sedentary lifestyles are increasingly common, as screen-based leisure activities replace active ones [10,16,22]. Conversely, participation in club sports increases the likelihood of meeting PA guidelines, especially vigorous PA, and supports the development of key physical, behavioral, and cognitive skills [23,24,25]. Importantly, sports participation during youth is often maintained into adulthood [26].

In 2020, the WHO updated its guidelines for children and adolescents (5–17 years), recommending an average of 60 min of moderate-to-vigorous PA per day and limiting sedentary time, especially recreational screen time (ST) [27,28]. Canadian guidelines similarly advise restricting daily ST to under two hours, with lower amounts conferring additional benefits—a threshold endorsed by other researchers [28,29,30] and the American Academy of Pediatrics [31]. Research on ST mainly focuses on mental health, highlighting associations between excessive ST and negative outcomes [32], but strong links with obesity have also been observed [33]. Overall, excessive ST appears to be associated with various health issues in young people, including obesity, an unhealthy diet, depressive symptoms, and a reduced quality of life [33].

Despite the recognized importance of active lifestyles, most adolescents do not achieve recommended PA levels. In Italy, 88.6% are inactive, with inactivity increasing from 2001 to 2016 in both sexes from 82.9% in 2001 to 85.9% in 2016 for boys and from 90.6% to 91.5% for girls [34]. Globally, over 80% of adolescents aged 11–17 do not meet the WHO’s PA recommendations, with higher rates in lower-income countries and with a progressive decline as age increases [27,34].

The COVID-19 pandemic further exacerbated these issues by reinforcing behaviors associated with unhealthy weight gain, such as reduced PA, increased ST, and higher caloric intake [35,36]. A systematic review found that ST among children and adolescents rose by 52% during the pandemic, with adolescents aged 12–18 showing an average increase of 110 min per day [37]. Social isolation and remote schooling contributed to rising obesity rates and related comorbidities [38,39]. In Italy, recent data indicate that 91.6% of individuals aged 15–17 use the internet daily, and only 15% of adolescents aged 11–19 do not have a social media profile [40,41].

Although several studies have examined the associations between PA, sedentary behavior, and adiposity in children and adolescents, the available evidence presents important limitations. Many investigations are cross-sectional, which limits causal interpretation [28,42], despite the recognized superiority of longitudinal approaches for identifying causal relationships [12,43,44,45]. Furthermore, they often rely exclusively on BMI, which cannot distinguish between fat and fat-free components during growth [46,47]. Longitudinal studies have reported inconsistent associations between ST, PA, and adiposity, with some showing weak or null effects, particularly when more detailed body composition measures are considered [47,48,49]. Moreover, few studies have focused on early adolescence, a period characterized by rapid and sex-specific changes in body composition, and even fewer have analyzed annual increments rather than absolute values. Consequently, it remains unclear whether participation in organized sports and screen-based sedentary behavior influence the trajectory of body composition changes during early adolescence, rather than cross-sectional outcomes alone. Addressing these gaps requires longitudinal designs, sex-specific analyses, and the use of multiple body composition indices beyond BMI.

In this context, the present study longitudinally investigates the relationship between lifestyle habits—specifically organized sports participation and screen device use—and multiple body composition parameters in Italian adolescents aged 11–13 years, analyzed by sex. Understanding how these behaviors influence body composition can guide targeted interventions to counteract declining PA levels and increasing screen exposure.

## 2. Materials and Methods

### 2.1. Research Design and Participants

A three-year longitudinal study was conducted on 158 Italian adolescents (98 boys and 60 girls; mean age at baseline: 11.3 ± 0.3 years) attending school years six to eight between November 2020 and November 2024. The same trained operators administered all of the assessments. The study took place in a public middle school in Ferrara (Emilia-Romagna, northern Italy), selected through convenience sampling.

Participants were recruited via official school letters requiring written informed consent from parents or guardians. All adolescents who provided consent were eligible and were invited to attend annual follow-up assessments, which included direct anthropometric measurements and a short questionnaire. Participants were informed that they could withdraw from the study at any time.

Sample size estimation was performed using G*Power (version 3.1.9.6; Universität Kiel, Germany). Assuming a medium effect size, 80% power, and α = 0.05, the minimum sample size required for sex comparisons (ANOVA on quantitative traits) was 128 participants. As suggested by Andrade [50], these primary analyses were employed to determine the a priori sample size.

The study was approved by the Bioethics University Committee (Ethical Approval Ref. no. 2.18).

### 2.2. Active and Inactive Behaviors

A specific dimension of PA, namely sport participation, and ST were assessed using brief self-report items, a pragmatic approach commonly adopted in large pediatric field studies to reduce participant burden (among others: [3,51,52]). Sedentary behaviour related to electronic device use was assessed with the questions: “Do you use screen devices?” and, if yes, “How many hours a day do you usually spend looking at a screen (television, computer, tablet, mobile phone, or gaming console)?”. Subsequently, taking into account the literature [29,53], we classified participants into two groups: ≤2 h/day and >2 h/day of ST.

Sport participation was assessed through a series of simple, study-specific questions, designed to capture engagement in structured and regular physical activity, rather than overall physical activity level. Organized sports participation was assessed through four questions: “Do you play any organized sports?”, “What is the main type of sport you play?”, “How many hours per week?”, and “At what age did you start?”

### 2.3. Anthropometric Measurements

Direct anthropometric measurements were taken by a single trained operator (L.Z.) according to standard procedures [54,55,56]. Measurements were performed in a private area of the school to ensure confidentiality.

Participants, barefoot and in light clothing, were weighed to the nearest 0.5 kg using a mechanical scale (SECA, Basel, Switzerland). Stature was measured to the nearest 0.1 cm using an anthropometer (Magnimeter, Raven Equipment Ltd., Essex, UK) while the adolescent was in a straightened position with the head oriented on the Frankfurt plane. Skinfold thicknesses (triceps and subscapular) were taken on the left side of the body, as suggested by Weiner and Lourie [54], using a Lange caliper (Beta Technology Inc., Houston, TX, USA) to the nearest 0.5 mm. Waist circumference was measured to the nearest 0.1 cm using a non-stretchable tape (DKSH, Zurich, Switzerland) at the midpoint between the lower margin of the last palpable rib and the top of the iliac crest [57].

Body fat was assessed using the skinfold method following standardized anthropo-metric procedures. Measurement reliability was ensured by a pre-study technical error of measurement (TEM) < 5% for skinfold thickness. The validity of skinfold-derived body fat estimates has been demonstrated through comparisons with reference methods such as dual-energy X-ray absorptiometry [58]. Body fat percentage (%F) was estimated using the equations by Slaughter et al. [59], applying the appropriate age-specific intercepts for boys [45,60]. Children aged under 12 were considered prepubescent, those aged 12–13.9 were considered pubescent, and those aged 14 and over were considered post-pubescent. Fat mass (FM, kg) and fat-free mass (FFM, kg) were derived accordingly. Fat mass index (FMI, kg/m^2^) and fat-free mass index (FFMI, kg/m^2^) were calculated as FM or FFM divided by stature squared, respectively.

Body mass index (BMI, kg/m^2^) was computed by dividing weight (kg) by stature squared (m^2^) and used to classify adolescents as underweight, normal weight, overweight, or obese according to age- and sex-specific cut-offs [61,62]. Finally, we calculated the waist-to-height ratio (WHtR) by dividing waist circumference by stature and categorized participants into three categories following established cutoffs [63,64] into normal central adiposity (WHtR < 0.50), increased central adiposity (0.5 ≤ WHtR < 0.6), and high central adiposity (WHtR ≥ 0.6).

Annual changes (increments) in body composition traits and indices were analyzed by sex.

### 2.4. Statistical Analysis

Baseline and follow-up descriptive statistics were reported as means and standard deviations (SDs) for continuous variables, stratified by sex and age group, and as absolute and relative frequencies for categorical variables. The Kolmogorov–Smirnov test was used to assess normality.

A repeated-measures ANOVA tested the effects of sex and age on quantitative variables at ages 11, 12, and 13. Effect sizes were calculated using partial eta squared (η^2^_p_) and interpreted based on Cohen’s conventional benchmarks (0.01 = small, 0.06 = medium, and 0.14 = large) [65]. Chi-squared tests were used to compare categorical variables between sexes.

Multiple linear regression analyses assessed associations between sex (coded 1 = male; 2 = female), behavioral variables (years of organized sports participation, current weekly hours of sports, and ST in h/day), and each body composition parameter (dependent variables). Multicollinearity was evaluated using the variance inflation factor (VIF), with values between 0.10 and 10 considered acceptable [66].

All analyses were performed using Statistica for Windows (version 11.0; StatSoft Srl, Tulsa, OK, USA). Statistical significance was set at *p* < 0.05.

## 3. Results

Table 1 shows the descriptive characteristics of the sample examined across the three survey years. From 11 to 13 years of age, participants showed the expected increases in stature, weight, and waist circumference, while changes in adipose tissue thicknesses differed by sex. In the first two surveys, girls were taller and heavier than boys, but by the third year, boys had reached and surpassed them. The sum of the skinfolds increased with age in females and decreased in males. Waist circumference was consistently lower in girls at every age.

Boys were much more likely than girls to participate in sports (*p* = 0.0158 in the first survey; *p* = 0.0647 in the second; *p* = 0.0003 in the third) and also reported more weekly hours of sports activities (*p* = 0.0050 in the first survey; *p* = 0.0825 in the second; *p* = 0.0010 in the third). Among boys, organized activities were predominantly team sports (70.7%), especially football (46.7%) and basketball (9.3%). Girls preferred individual sports (67.6%), particularly dance (36%) and swimming (28%). Volleyball was the most practiced team sport among girls (28%), whereas swimming was the main individual activity for boys (17%).

ST increased with age, especially among girls. Device use was very high in both sexes and reached 100% among 13-year-old girls. Girls spent significantly more hours per day in front of screens in the second (*p* = 0.0027) and third year (*p* = 0.0339).

Table 2 presents sex comparisons for body composition parameters and indices and their annual increments. Beyond significant effects of repeated measures and sex, the repeated-measures ANOVA revealed a significant interaction between survey year and sex for eight variables (%F, FM, FFM, FFM increment, WHtR, WHtR increment, FMI, and FFMI). These interaction effects were generally of small to moderate magnitude (η^2^_p_ ≈ 0.03–0.24), with the largest effects observed for FFM (η^2^_p_ = 0.24) and FFM increment (η^2^_p_ = 0.10). Overall, body size increased across the three years, with boys showing greater gains—particularly in FFM and FFMI. The main effect of sex was significant for %F, %F increment, FM increment (all higher in girls), as well as for FFM increment, BMI increment, WHtR and WHtR increment, FMI, and FFMI increment. The magnitude of sex effects ranged from small to moderate (η^2^_p_ ≈ 0.02–0.12), with the largest effect observed for %F increment (η^2^_p_ = 0.12) and FFM increment (η^2^_p_ = 0.28), indicating meaningful sex-related differences in body composition changes over time.

Across the sample, most adolescents fell within the normal weight range (boys: 64.3%; girls: 67.8%) and showed normal levels of central adiposity (boys: 81.7%; girls: 92.8%). On average, 30.3% of boys and 28.3% of girls were overweight or obese, while increased or high central adiposity was observed in 18.3% of boys and 7.2% of girls. Variations in weight status and central adiposity over the three years were not statistically significant. Between-sex comparisons revealed a significant difference only at age 11 for central adiposity (*p* = 0.023), with girls showing a higher prevalence of normal levels and a lower prevalence of increased adiposity.

Statistical differences in weight status and central obesity according to ST and sport participation by age and sex are reported in Table 3 and Table 4, respectively, while the corresponding distributions are shown in Supplementary Materials (Table S1 and Figures S1–S8).

No significant differences emerged between behavioural categories (ST ≤ 2 h/day vs. >2 h/day; sport vs. no sport) in either sex.

A chi-square test comparing sexes within the inactive group (no sport) showed a significant difference at age 11 only (*p* = 0.0467), with boys displaying a higher incidence of increased central adiposity than girls.

Finally, multiple regression analyses were used to assess whether sports and ST habits could explain body composition parameters and indices. VIF values ranged from 1 to 1.2, indicating no collinearity among predictors. Only significant models (Table 5) are reported and refer to 13-year-olds. The models for increments in %F, FFM, WHtR, and FMI explained between 6% and 21% of variance. Sex emerged as a significant determinant of changes in %F and WHtR (positively) and FFM (negatively), while weekly hours of sport were significant predictors of increments in %F and FMI (negatively).

## 4. Discussion

Physical inactivity in children and adolescents increases the risk of several health problems, including obesity, cardiovascular disease, and mental disorders (among others: [12,67,68]). The present longitudinal study provides new insights into body composition changes in Italian adolescents aged 11 to 13 years, with a specific focus on the associations between organized sports participation, ST, and annual increments in multiple body composition parameters. By analyzing annual increments rather than cross-sectional values alone, this study provides a more dynamic perspective on growth-related processes occurring during early adolescence, a period characterized by marked biological and behavioral transitions [2,69].

The main results obtained will be discussed by addressing the salient points and comparing them with those of other studies. This will be done despite the difficulties created by differences in measurement protocols for both PA and body composition, as well as the frequent lack of sex-stratified analyses, which complicate the outcomes reported in the existing literature. Furthermore, a few studies have examined body changes and sedentary behavior.

### 4.1. Sex Differences and Growth-Related Changes

As expected, clear sex-specific patterns emerged in body composition trajectories. Boys showed larger increases in FFM and FFMI over time, whereas girls exhibited higher levels and greater increments in %F and fat-mass-related indices. These findings reflect the well-documented differences in pubertal timing and hormonal changes. Girls generally enter and progress through puberty earlier than boys, experiencing oestrogen-driven increases in adiposity, while boys undergo later but more pronounced testosterone-related gains in FFM [70,71]. The absence of direct measures of pubertal status prevents a more precise disentanglement of biological maturation from behavioral effects. Nevertheless, the longitudinal analysis of annual changes provides indirect insight into developmental processes occurring during this critical period. Incorporating pubertal staging or maturation indicators (e.g., Tanner stages or age at peak height velocity) into future research could allow for a more refined interpretation of the interplay between lifestyle behaviors and growth-related changes.

Previous longitudinal studies [72,73,74] have similarly reported sex-specific changes in adiposity and lean mass during this developmental window, highlighting the importance of stratifying analyses by sex when investigating growth and body composition outcomes.

### 4.2. Organized Sports Participation and Body Composition Trajectories

A key finding of this study is that organized sports participation was not associated with cross-sectional body composition parameters, but the number of weekly hours spent practicing sports emerged as a significant predictor of annual increments in fat-related indices at the age of 13. Specifically, greater involvement in sports was associated with smaller increases in body %F and FMI. This apparent discrepancy between cross-sectional and longitudinal findings may help explain the inconsistent results reported in previous research on the relationships between sports participation and adiposity in young people. Despite the expected link between exercise and lower adiposity, this study did not find a direct influence of sports participation on the main body composition parameters and indices, as reported in other studies on younger children [75] and adolescents [45,48], suggesting that the relationship between organized sports and adiposity may be weak or absent throughout adolescence. However, unlike previous work, the calculation of annual increments revealed that weekly hours of sports practice significantly influenced changes in %F and FMI at age 13, indicating that such null findings may reflect the limited sensitivity of cross-sectional designs and single-time-point indicators to capture gradual behavioral effects. Therefore, the present results suggest that organized sports may influence the direction and pace of body composition changes rather than producing large differences in absolute adiposity at early adolescence.

From a developmental perspective, early adolescence is a critical period during which relatively minor behavioral influences may shape long-term growth trajectories. Regular participation in organized sports can help to reduce fat accumulation over time and promote greater lean mass development [76,77]. While these effects are likely to be modest at the individual level, they may become more significant when considered cumulatively across adolescence.

### 4.3. Screen Time and Sedentary Behavior

Unlike sports participation, ST was not significantly associated with cross-sectional body composition parameters or their annual changes. A recent review suggests that high ST may increase the risk of overweight and obesity in children and adolescents [78]. Our data, however, do not support the idea that a two-hour ST cut-off represents a risk threshold in early adolescence. No differences in weight status were detected according to ST, contrasting with the cross-sectional findings of Nagata et al. [42]. One possible explanation is the high prevalence of ST device use in the present sample, particularly among 13-year-olds, which may have limited variability and reduced the discriminatory power of the commonly used two-hour cut-off. Furthermore, the lack of significant associations between ST and body composition parameters should be interpreted with caution, as several unmeasured factors may have contributed to these null findings. Dietary intake, sleep duration and quality, and socioeconomic status are known to interact with sedentary behaviors and influence adiposity during adolescence [45,79,80]. Still, these factors were not assessed in the present study. The absence of these variables may have resulted in residual confounding factors, which could have obscured more complex relationships between ST and body composition. However, our finding is consistent with the results of several longitudinal studies [46,47], which have reported weak or null associations between sedentary behavior and adiposity when ST is considered independently of overall PA levels.

Measurement-related issues should also be considered. ST was assessed using simple self-reporting and did not differentiate between types of screen-based activity, such as watching television, passively using a smartphone, or actively playing video games. There is emerging evidence [78,81] that different types of screen use may be associated with different patterns of PA and energy expenditure, which a single aggregate measure cannot capture. Finally, high levels of ST do not necessarily imply physical inactivity, since adolescents may also participate in organized sports or other moderate-to-vigorous PA. This behavioral compensation could therefore reduce the effect of ST on body composition.

Regarding the collection of ST data through simple questions and its subsequent dichotomization in relation to the cut-off point, this study adopted a pragmatic approach that is commonly used in school-based research on children and adolescents. Brief self-report items for screen-based sedentary behaviors have been shown to provide acceptable reliability in youth populations for large-scale observational studies [82,83], and the use of a 2 h daily threshold aligns with international sedentary behavior guidelines and recommendations of the American Academy of Pediatrics [31,84]. However, this approach may have reduced measurement sensitivity and weakened the strength of the observed associations. In particular, the dichotomization of ST according to the 2 h/day guideline, although aligned with international recommendations, may have obscured potential dose–response relationships and limited the detection of more nuanced behavioral effects. Consequently, the reported associations should be considered conservative estimates, and future studies would benefit from more detailed and objective assessments of PA and sedentary behavior.

### 4.4. Interpreting Regression Models and Explained Variance

The multivariate models obtained confirmed the existence of several significant associations. Although the regression models explained a relatively small proportion of the variance in body composition increments, this finding is not unexpected in studies examining behavioral determinants of growth-related outcomes. Changes in body composition during early adolescence are shaped by complex and interacting biological processes, including pubertal timing, hormonal changes, genetic predisposition, and environmental factors, which were not fully captured in the present models. Lifestyle behaviors, such as participation in sports and ST, represent only one component of this multifactorial process. In this context, the observed associations should be interpreted as modest yet meaningful contributions, rather than strong explanatory models. The fact that organized sports participation remained a significant predictor of fat-related increments despite low R^2^ values suggests that behavioral factors may have subtle yet important effects on developmental trajectories. In further detail, more hours of sport were associated with smaller annual increments in %F and FMI. Being female was associated with larger increments in %F and WHtR and smaller increments in FFM. In contrast to the findings of Mitchell et al. [85], who found an association between sedentary behavior and higher BMI percentiles in children aged 9–15, our results indicate that ST was never a significant determinant, approaching significance only for the increment in FFM (negative association).

Overall, the multivariate regression analysis provides new evidence that participation in sports is associated with annual changes in body composition parameters at age 13. These findings underscore the importance of promoting organized sports during adolescence to support healthier growth trajectories and prevent the development of unhealthy body composition patterns and weight in adulthood.

### 4.5. Strengths and Limitations

The main strengths of this study are its longitudinal design, the repeated direct measurement of anthropometric variables by a trained operator. Another strength is the use of multiple indices of adiposity beyond BMI, including WHtR, which is considered a more appropriate measure for adolescents undergoing growth than waist circumference alone [86]. The analysis of annual increments represents an additional methodological strength, allowing the detection of changes that may be obscured in cross-sectional analyses. Several limitations should be acknowledged. The use of a convenience sample with a preponderance of boys and recruited from a single school limits the generalizability of the findings. Consequently, the results should not be interpreted as population-level estimates, but rather as evidence derived from a longitudinal study aimed at exploring associations and developmental trajectories. Nevertheless, the homogeneous school setting and repeated assessments by the same trained operator strengthen internal validity and reduce measurement variability.

Moreover, lifestyle behaviors were assessed using brief self-report items, which, while pragmatic and feasible in school-based studies, provide limited detail. In particular, information on sport type and training load, and specific types of screen-based activities, was not collected or used in multiple regression analysis. However, as concerns the sport type, we should emphasise that it does not appear to affect body composition during the early adolescent period according to the literature [48]. Anyway, this limited granularity may have resulted in non-differential misclassification and an attenuation of the observed associations. Furthermore, ST was dichotomised according to the ≤2 h/day guideline threshold. Although this approach aligns with current recommendations and facilitates interpretation, it may obscure dose–response relationships and reduce variability, potentially limiting the ability to detect stronger associations.

Additionally, a lack of information on diet, sleep, and pubertal stage potentially limited the explanatory power of the models. In particular, indirectly assessing pubertal status and failing to consider differences in the timing of puberty may have affected sex-specific body composition patterns and annual increments. Nevertheless, the homogeneous school setting and consistent measurement procedures enhance the models’ internal validity and reduce measurement variability.

Future studies should consider using more detailed instruments or objective measures to capture the different PA levels and screen-based behaviors.

## 5. Conclusions

In conclusion, we found no clear evidence that extracurricular sports participation or ST between ages 11 and 13 is directly associated with body composition parameters or indices. However, by analyzing annual increments rather than cross-sectional values alone, our study was able to detect subtle yet meaningful changes in body composition that would otherwise have remained hidden. This provides evidence that organized sports participation during early adolescence is associated with more favorable trajectories of fat-related body composition indices, even in the absence of strong cross-sectional associations. These longitudinal increments proved particularly informative at age 13, when multivariate models showed that adiposity changes were significantly associated with behavioral factors—especially weekly hours of sports practice—alongside sex. The lower increments in %F and FMI observed in adolescents who practiced more hours of sport per week highlight the added value of longitudinal assessment in identifying emerging patterns in growth and adiposity. This approach enables more accurate evaluation of the influence of lifestyle factors on developmental trajectories during early adolescence, a period characterized by rapid and heterogeneous biological changes, underscoring the importance of adopting longitudinal approaches and analyzing annual increments when investigating the impact of lifestyle behaviors on adolescent growth.

Promoting regular sports participation during early adolescence is fundamental, not only for its immediate benefits, but also for its long-term contribution to healthier developmental trajectories. Future initiatives should encourage healthy habits by promoting PA and offering structured opportunities for adolescents to engage in organized sports, while also monitoring and moderating daily ST.

## Figures and Tables

**Table 1:** Anthropometric and behavioural characteristics of boys (n = 98) and girls (n = 60) aged 11–13 years.

Variables	Boys			Girls		
	11 Years	12 Years	13 Years	11 Years	12 Years	13 Years
	Mean ± SD	Mean ± SD	Mean ± SD	Mean ± SD	Mean ± SD	Mean ± SD
*Anthropometric traits*						
Stature (cm)	148.2 ± 6.8	155.0 ± 7.6	162.7 ± 7.9	151.0 ± 7.1	156.0 ± 6.5	158.6 ± 6.1
Weight (kg)	42.8 ± 9.3	48.8 ± 10.6	54.6 ± 11.3	45.1 ± 7.9	50.6 ± 9.1	53.9 ± 9.6
Waist circumference (cm)	67.2 ± 8.2	70.6 ± 9.1	72.1 ± 8.8	65.1 ± 6.3	67.5 ± 7.2	68.7 ± 7.4
Triceps skinfold (mm)	14.2 ± 5.9	14.5 ± 6.4	13.1 ± 6.3	15.5 ± 5.0	16.5 ± 5.1	17.8 ± 6.1
Subscapular skinfold (mm)	11.2 ± 6.6	12.0 ± 6.8	11.7 ± 6.7	12.4 ± 5.5	13.0 ± 5.9	14.1 ± 7.3
Sum of two skinfolds (mm)	25.3 ± 11.9	26.5 ± 12.6	24.8 ± 12.7	27.9 ± 9.9	29.5 ± 10.5	31.9 ± 12.9
*Behavioural traits*						
Sports practice (YES) n (%)	75 (76.5)	74 (75.5)	79 (80.6)	35 (58.3)	37 (61.7)	32 (53.3)
Years of sports practice	3.9 ± 2.1	3.9 ± 2.6	4.0 ± 2.9	3.7 ± 1.8	3.5 ± 2.4	4.1 ± 2.9
Current sports amount (h/week)	3.4 ± 2.9	3.3 ± 2.7	4.1 ± 2.8	2.2 ± 2.4	2.5 ± 2.9	2.5 ± 3.3
Use of screens (YES) n (%)	89 (90.8)	95 (96.9)	96 (98.0)	54 (90.0)	59 (98.3)	60 (100)
Screen time (h/day)	2.4 ± 1.5	2.3 ± 1.4	2.9 ± 1.9	2.3 ± 1.8	3.0 ± 1.5	3.6 ± 2.1

Note: values are expressed as mean ± standard deviation or n (%), as appropriate.

**Table 2:** Body composition parameters, indices, and related annual increments according to sex, analyzed using ANOVA for repeated measures (males n = 98; females n = 60).

	Boys			Girls			ANOVA					
Variables	11 Years	12 Years	13 Years	11 Years	12 Years	13 Years	Sex		Measures		Sex*Measures	
	Mean ± SD	Mean ± SD	Mean ± SD	Mean ± SD	Mean ± SD	Mean ± SD	*p*	η^2^_p_	*p*	η^2^_p_	*p*	η^2^_p_
% F	22.93 ± 9.19	22.54 ± 10.05	20.91 ± 10.23	23.69 ± 6.44	24.90 ± 6.50	26.38 ± 7.66	**0.04**	0.03	0.6	0	**<0.0001**	0.09
% F increment (%/year)	-	−0.39 ± 5.19	−1.63 ± 5.10	-	1.21 ± 4.02	1.48 ± 4.20	**<0.0001**	0.12	0.42	0	0.21	0.01
FM (kg)	10.52 ± 6.75	11.79 ± 7.53	12.17 ± 8.06	11.00 ± 4.49	12.97 ± 5.32	14.68 ± 6.53	0.19	0.01	**<0.0001**	0.21	**0.003**	0.04
FM increment (kg/year)	-	1.27 ± 3.18	0.39 ± 3.28	-	1.97 ± 2.69	1.71 ± 3.49	**0.005**	0.05	0.13	0.01	0.42	0
FFM (kg)	32.28 ± 4.18	37.04 ± 5.55	42.43 ± 6.88	34.11 ± 4.77	37.61 ± 5.27	39.22 ± 5.53	0.75	0	**<0.0001**	0.73	**<0.0001**	0.24
FFM increment (kg/year)	-	4.76 ± 2.61	5.39 ± 3.35	-	3.50 ± 2.31	1.60 ± 1.91	**<0.0001**	0.28	**0.036**	0.03	**<0.0001**	0.1
BMI (kg/m^2^)	19.37 ± 3.21	20.19 ± 3.41	20.51 ± 3.40	19.72 ± 2.80	20.76 ± 3.37	21.39 ± 3.42	0.25	0.01	**<0.0001**	0.35	0.06	0.02
BMI increment (kg/m^2^/year)	-	0.82 ± 1.13	0.32 ± 0.97	-	1.04 ± 1.48	0.63 ±1.21	**0.05**	0.02	**0.001**	0.06	0.74	0
WHtR	0.453 ± 0.050	0.456 ± 0.055	0.443 ± 0.052	0.432 ± 0.044	0.433 ± 0.049	0.433 ± 0.048	**0.02**	0.03	**0.01**	0.03	**0.002**	0.04
WHtR increment (cm/cm/year)	-	0.002 ± 0.026	−0.012 ± 0.021	-	0.001 ± 0.022	0.0004 ± 0.0183	**0.009**	0.04	**0.01**	0.04	**0.023**	0.03
FMI (kg/m^2^)	4.70 ± 2.73	4.84 ± 2.91	4.57 ± 2.94	4.82 ± 1.96	5.35 ± 2.25	5.84 ± 2.60	0.13	0.02	**<0.0001**	0.05	**<0.0001**	0.07
FMI increment (kg/m^2^/year)	-	0.14 ± 1.34	−0.28 ± 1.28	-	0.53 ± 1.14	0.49 ± 1.38	**<0.0001**	0.1	0.15	0.01	0.23	0.01
FFMI (kg/m^2^)	14.66 ± 1.19	15.35 ± 1.40	15.95 ± 1.68	14.90 ± 1.31	15.41 ± 1.50	15.55 ± 1.67	0.89	0	**<0.0001**	0.33	**0.0002**	0.05
FFMI increment (kg/m^2^/year)	-	0.68 ± 0.80	0.60 ± 1.00	-	0.51 ± 0.86	0.14 ± 0.74	**0.0007**	0.07	**0.04**	0.03	0.19	0.01

Note: Bold indicates significant values; η^2^_p_ = partial eta squared; BMI = body mass index; %F = body fat percentage; FM = fat mass; FMI = fat mass index; FFM = fat free mass; FFMI = fat free mass index; WHtR = waist-to height ratio.

**Table 3:** Statistical significance of differences in weight status according to sedentary time (ST ≤ 2 h/day vs. >2 h/day) and sport participation (Sport vs. No sport) by age and sex (males n = 98; females n = 60).

Age (Years)	Sex	Comparison	*p*-Value
11	Boys	ST ≤ 2 h/day vs. >2 h/day	0.241
11	Girls	ST ≤ 2 h/day vs. >2 h/day	0.911
11	Boys	Sport vs. No sport	0.118
11	Girls	Sport vs. No sport	0.952
12	Boys	ST ≤ 2 h/day vs. >2 h/day	0.896
12	Girls	ST ≤ 2 h/day vs. >2 h/day	0.995
12	Boys	Sport vs. No sport	0.283
12	Girls	Sport vs. No sport	0.636
13	Boys	ST ≤ 2 h/day vs. >2 h/day	0.666
13	Girls	ST ≤ 2 h/day vs. >2 h/day	0.979
13	Boys	Sport vs. No sport	0.982
13	Girls	Sport vs. No sport	0.906

Note: *p*-values were derived from chi-square tests. Detailed age- and sex-specific distributions of weight status are reported in the Supplementary Materials (Table S1).

**Table 4:** Statistical significance of differences in central obesity according to sedentary time (ST ≤ 2 h/day vs. >2 h/day) and sport participation (Sport vs. No sport) by age and sex (males n = 98; females n = 60).

Age (Years)	Sex	Comparison	*p*-Value
11	Boys	ST ≤ 2 h/day vs. >2 h/day	0.463
11	Girls	ST ≤ 2 h/day vs. >2 h/day	1
11	Boys	Sport vs. No sport	0.058
11	Girls	Sport vs. No sport	0.368
12	Boys	ST ≤ 2 h/day vs. >2 h/day	0.588
12	Girls	ST ≤ 2 h/day vs. >2 h/day	0.858
12	Boys	Sport vs. No sport	0.11
12	Girls	Sport vs. No sport	0.536
13	Boys	ST ≤ 2 h/day vs. >2 h/day	0.808
13	Girls	ST ≤ 2 h/day vs. >2 h/day	0.32
13	Boys	Sport vs. No sport	0.3
13	Girls	Sport vs. No sport	0.89

Note: *p*-values were derived from chi-square tests. Detailed age- and sex-specific distributions of weight status are reported in the Supplementary Materials (Table S1).

**Table 5:** Predictors of body composition increments by multivariate regression analysis in 13-year-old adolescents.

	% F Increment			FFM Increment			WHtR Increment			FMI Increment		
	β	t	*p*	β	t	*p*	β	t	*p*	β	t	*p*
Sex (reference: female)	0.188	2.026	**0.045**	−0.421	−4.945	**0.000003**	0.323	3.556	**0.0005**	0.170	1.833	0.070
Years of sports practice	−0.007	−0.065	0.948	−0.031	−0.339	0.735	−0.044	−0.450	0.654	−0.025	−0.245	0.807
Sport hours	−0.204	−2.048	**0.043**	0.139	1.514	0.133	−0.121	−1.240	0.218	−0.227	−2.268	**0.025**
Screen time	0.113	1.224	0.224	−0.152	−1.788	0.077	0.041	0.447	0.656	0.068	0.733	0.465
												
*R* ^2^	0.1007			0.2397			0.1351			0.0977		
*Adjusted R* ^2^	0.0671			0.2113			0.1027			0.0640		
*p-value*	**0.0218**			**0.000006**			**0.0031**			**0.0252**		

Note: Bold indicates significant values.

## Data Availability

Data are available upon request due to ethical restrictions regarding participant privacy. Requests for the data may be sent to the corresponding author.

## Supplementary Materials

The following supporting information can be downloaded at: https://www.mdpi.com/article/10.3390/children13010130/s1, Table S1: Distribution of weight status and central obesity categories by screen time (ST ≤ or >2 h/day) and sports practice (yes or no): sex comparison by chi-squared test (males n = 98; females n = 60). Figure S1. Distribution of weight status categories according to screen time (≤2 vs. >2 h/day) across ages (11–13 years) in boys. Data are expressed as percentages. Figure S2. Distribution of weight status categories according to screen time (≤2 vs. >2 h/day) across ages (11–13 years) in girls. Data are expressed as percentages. Figure S3. Distribution of weight status categories according to sports practice (yes/no) across ages (11–13 years) in boys. Data are expressed as percentages. Figure S4. Distribution of weight status categories according to sports practice (yes/no) across ages (11–13 years) in girls. Data are expressed as percentages. Figure S5. Distribution of central obesity categories according to screen time (≤2 vs. >2 h/day) across ages (11–13 years) in boys. Data are expressed as percentages; Figure S6. Distribution of central obesity categories according to screen time (≤2 vs. >2 h/day) across ages (11–13 years) in girls. Data are expressed as percentages. Figure S7. Distribution of central obesity categories according to sports practice (yes/no) across ages (11–13 years) in boys. Data are expressed as percentages. Figure S8. Distribution of central obesity categories according to sports practice (yes/no) across ages (11–13 years) in girls. Data are expressed as percentages.

## Abbreviations

The following abbreviations are used in this manuscript:

ANOVA	Analysis of Variance
BMI	Body Mass Index
COVID-19	Coronavirus Disease 2019
%F	Body fat percentage
FFM	Fat-Free Mass
FFMI	Fat-Free Mass Index
FM	Fat Mass
FMI	Fat Mass Index
PA	Physical Activity
ST	Screen time
VIF	Variance inflation factor
WHtR	Waist-to-height ratio
