# Supplementary material for: Body Composition Changes and Their Associations with Physical Activity and Screen Time in a Sample of Italian Early Adolescents over a 3-Year Period
Source: Children (Basel). 2026 Jan 15;13(1):130. doi: 10.3390/children13010130 (PMC12839566; doi:10.3390/children13010130)
Supplement: Supplementary file 1 [file children-13-00130-s001.zip › children-4086948-supplementary.pdf]

Table S1. Distribution of weight status and central obesity categories by screen time (ST ≤ or > 2 hours/day) and sports practice (yes or no): sex comparison by chi-squared test (males n =98; females n = 60).

	Boys		Chi-square	Girls		Chi-square	Boys		Chi-square	Girls		Chi-square
	**ST ≤ 2 h/d**	**ST >2 h/d**		**ST ≤ 2 h/d**	**ST > 2 h/d**		**SPORT**	**NO SPORT**		**SPORT**	**NO SPORT**	
11 years	**N (%)**	**N (%)**	*p-value*	**N (%)**	**N (%)**	*p-value*	**N (%)**	**N (%)**	*p-value*	**N (%)**	**N (%)**	*p-value*
Weight Status			0.241			0.911			0.118			0.952
Underweight	2 (3.7)	3 (6.8)		2 (5.0)	-		5 (6.7)	-		1 (2.9)	1 (4.0)	
Normal weight	41 (75.9)	25 (56.8)		25 (62.5)	15 (75.0)		54 (72.0)	12 (52.2)		24 (68.6)	16 (64.0)	
Overweight	9 (16.7)	12 (27.3)		12 (30.0)	5 (25.0)		13 (17.3)	8 (34.8)		9 (25.7)	8 (32.0)	
Obese	2 (3.7)	4 (9.1)		1 (2.5)	-		3 (4.0)	3 (13.0)		1 (2.9)	0	
Central obesity			0.463			1.000			0.058			0.368
Normal adiposity	46 (85.2)	35 (79.5)		38 (95.0)	19 (95.0)		65 (86.7)	16 (69.6)		34 (97.1)	23 (92.0)	
Increased adiposity	8 (14.8)	9 (20.5)		2 (5.0)	1 (5.0)		10 (13.3)	7 (30.4)		1 (2.9)	2 (8.0)	
12 years												
Weight Status			0.896			0.995			0.283			0.636
Underweight	4 (6.0)	2 (6.5)		-	2 (5.3)		5 (6.8)	1 (4.2)		1 (2.7)	1 (4.3)	
Normal weight	42 (62.7)	17 (54.8)		17 (77.3)	27 (71.1)		46 (62.2)	13 (54.2)		29 (78.4)	15 (65.2)	
Overweight	18 (26.9)	10 (32.3)		4 (18.2)	7 (18.4)		21 (28.4)	7 (29.2)		5 (13.5)	6 (26.1)	
Obese	3 (4.5)	2 (6.5)		1 (4.5)	2 (5.3)		2 (2.7)	3 (12.5)		2 (5.4)	1 (4.3)	
Central obesity			0.588			0.858			0.110			0.536
Normal adiposity	53 (79.1)	23 (74.2)		20 (90.9)	34 (89.5)		60 (81.1)	16 (66.7)		34 (91.9)	20 (86.9)	
Increased adiposity	14 (20.9)	7 (22.6)		2 (9.1)	4 (10.5)		13 (17.6)	8 (33.3)		3 (8.1)	3 (13.1)	
High adiposity	-	1 (3.2)		-	-		1 (1.4)	-				
13 years												
Weight Status			0.666			0.979			0.982			0.906
Underweight	2 (4.8)	3 (5.4)		-	3 (7.0)		4 (5.1)	1 (5.6)		1 (3.1)	2 (7.1)	
Normal weight	30 (71.4)	34 (60.7)		11 (64.7)	27 (62.8)		52 (65.8)	12 (66.7)		21 (65.6)	17 (60.7)	
Overweight	10 (23.8)	15 (26.8)		5 (29.4)	10 (23.3)		20 (25.3)	4 (22.2)		8 (25.0)	7 (25.0)	
Obese	-	4 (7.1)		1 (5.9)	3 (7.0)		3 (3.8)	1 (5.6)		2 (6.3)	2 (7.1)	
Central obesity			0.808			0.320			0.300			0.890
Normal adiposity	36 (85.7)	47 (83.9)		15 (88.2)	41 (95.3)		69 (87.3)	14 (77.8)		30 (93.7)	26 (92.9)	
Increased adiposity	6 (14.3)	9 (16.1)		2 (11.8)	2 (4.7)		10 (12.7)	4 (22.2)		2 (6.3)	2 (7.1)	

Note: Bold indicates significant values; ST=screen time.

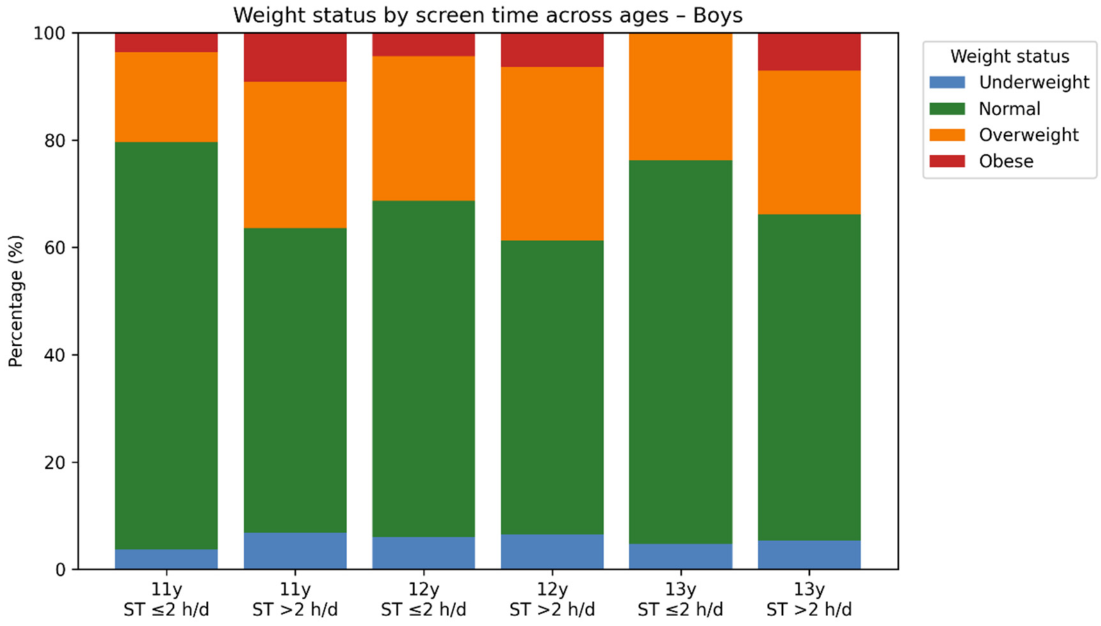

Figure S1. Distribution of weight status categories according to screen time (≤2 vs >2 h/day) across ages (11-13 years) in boys. Data are expressed as percentages.

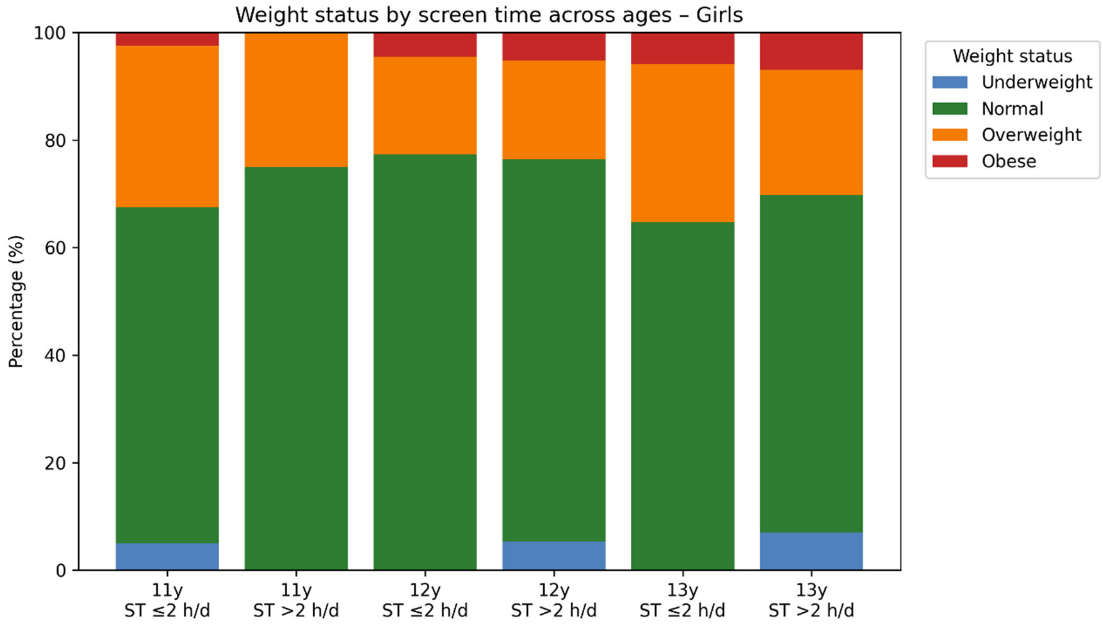

Figure S2. Distribution of weight status categories according to screen time (≤2 vs >2 h/day) across ages (11–13 years) in girls. Data are expressed as percentages.

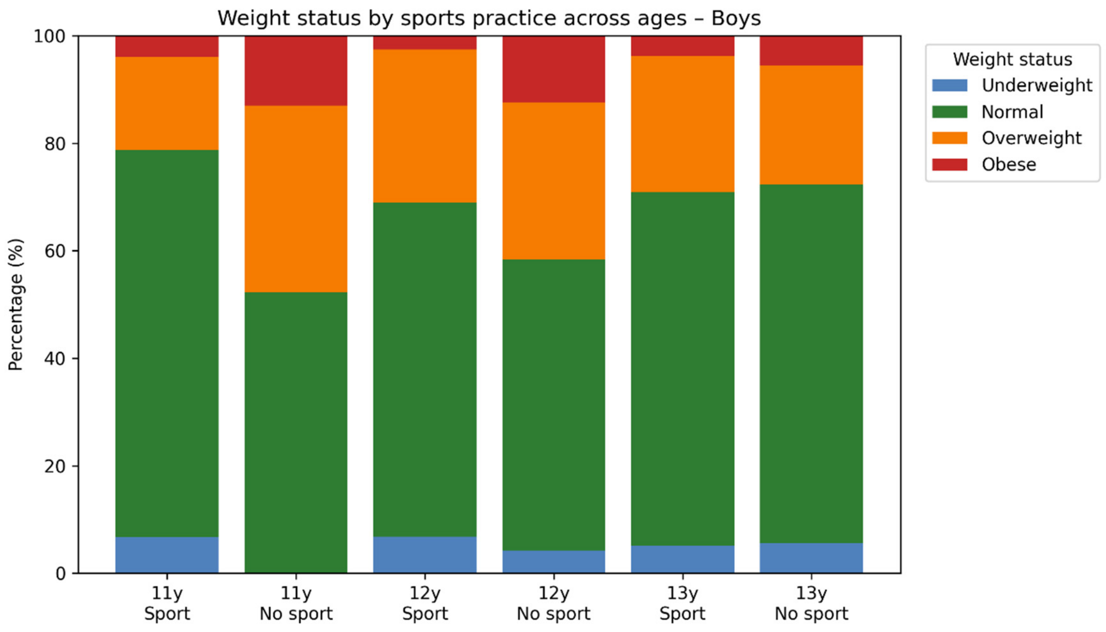

Figure S3. Distribution of weight status categories according to sport participation (sport vs > no sport) across ages (11–13 years) in boys. Data are expressed as percentages.

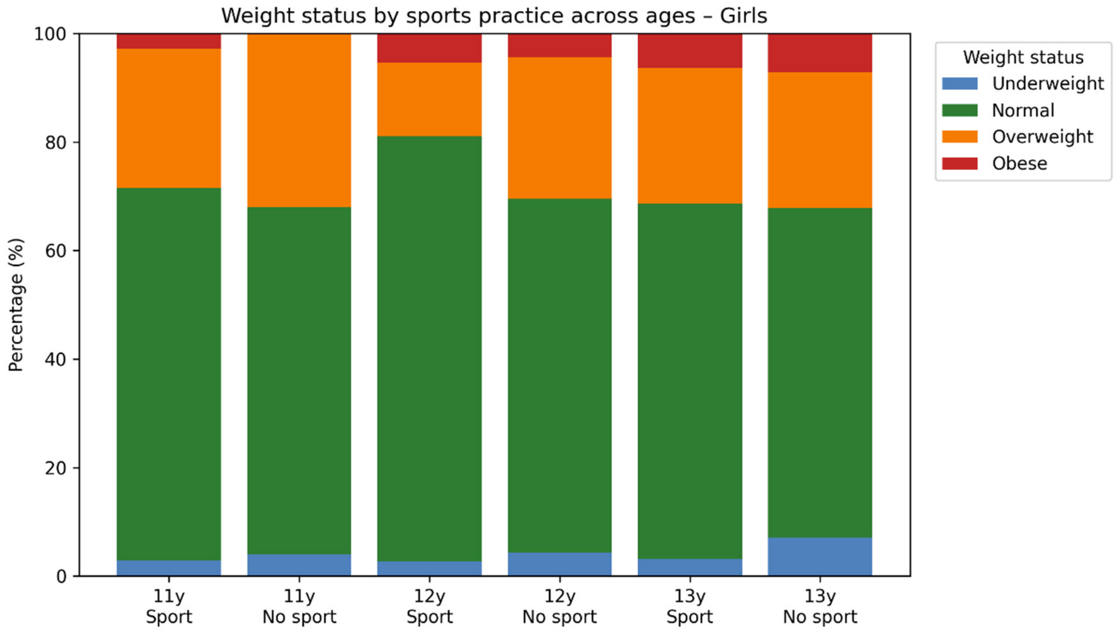

Figure S4. Distribution of weight status categories according to sport participation (sport vs > no sport) across ages (11–13 years) in girls. Data are expressed as percentages.

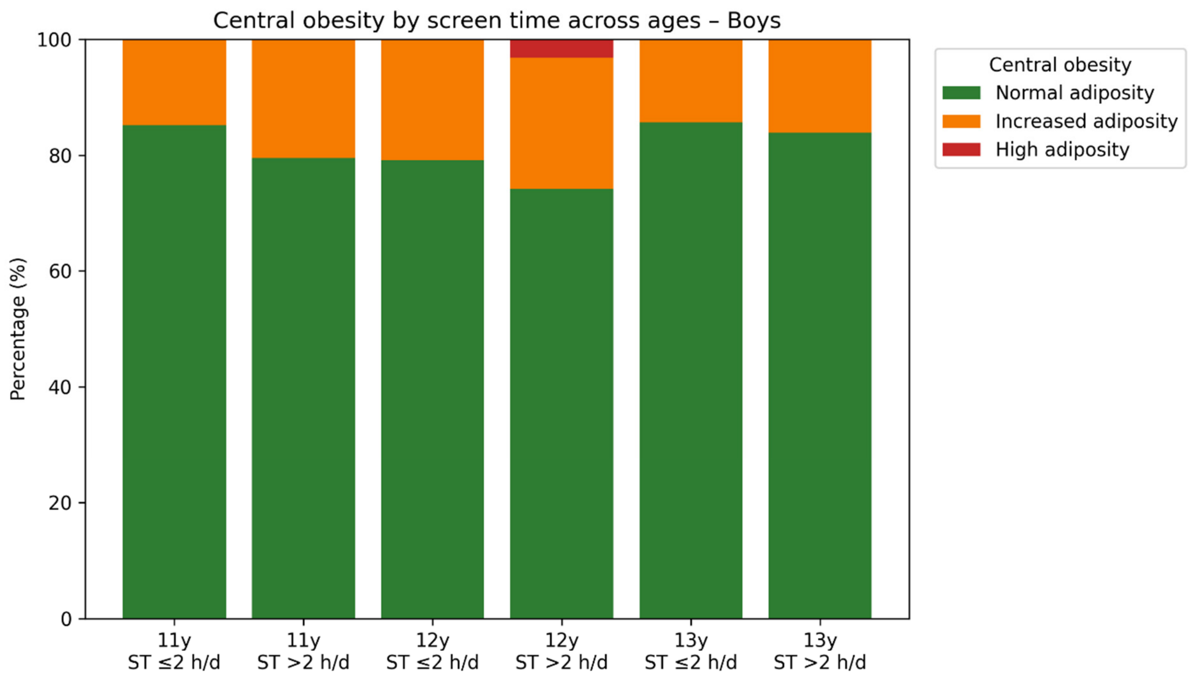

Figure S5. Distribution of central obesity categories according to screen time (≤2 vs >2 h/day) across ages (11–13 years) in boys. Data are expressed as percentages.

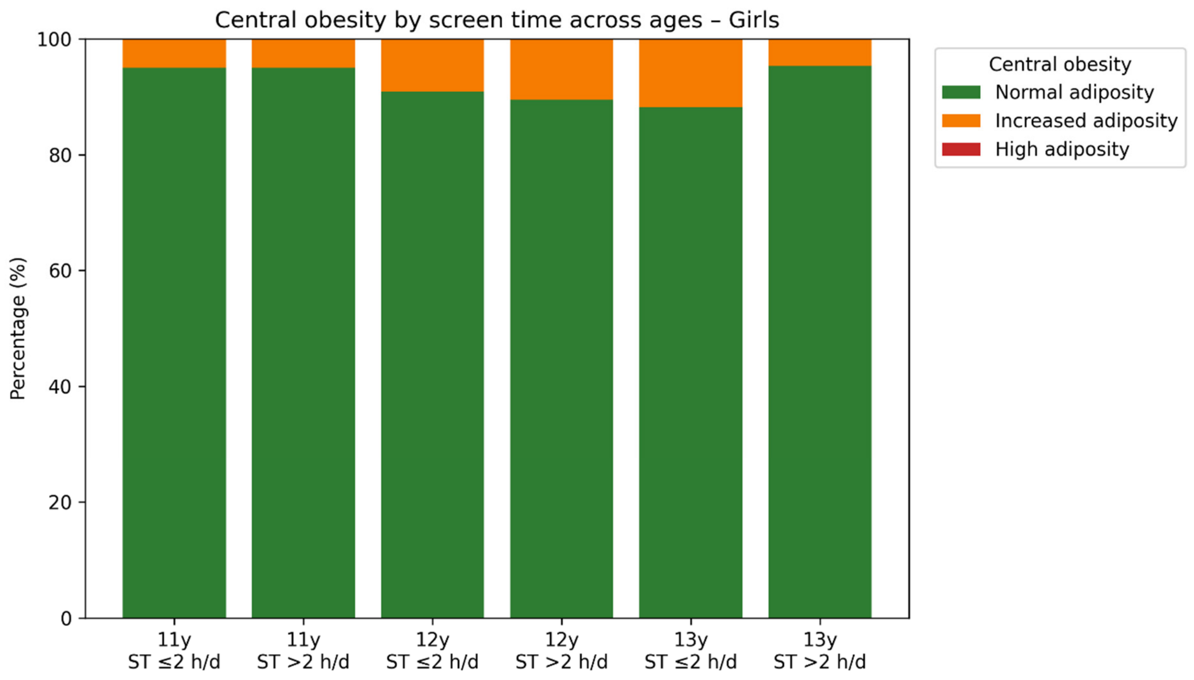

Figure S6. Distribution of central obesity categories according to screen time (≤2 vs >2 h/day) across ages (11–13 years) in girls. Data are expressed as percentages.

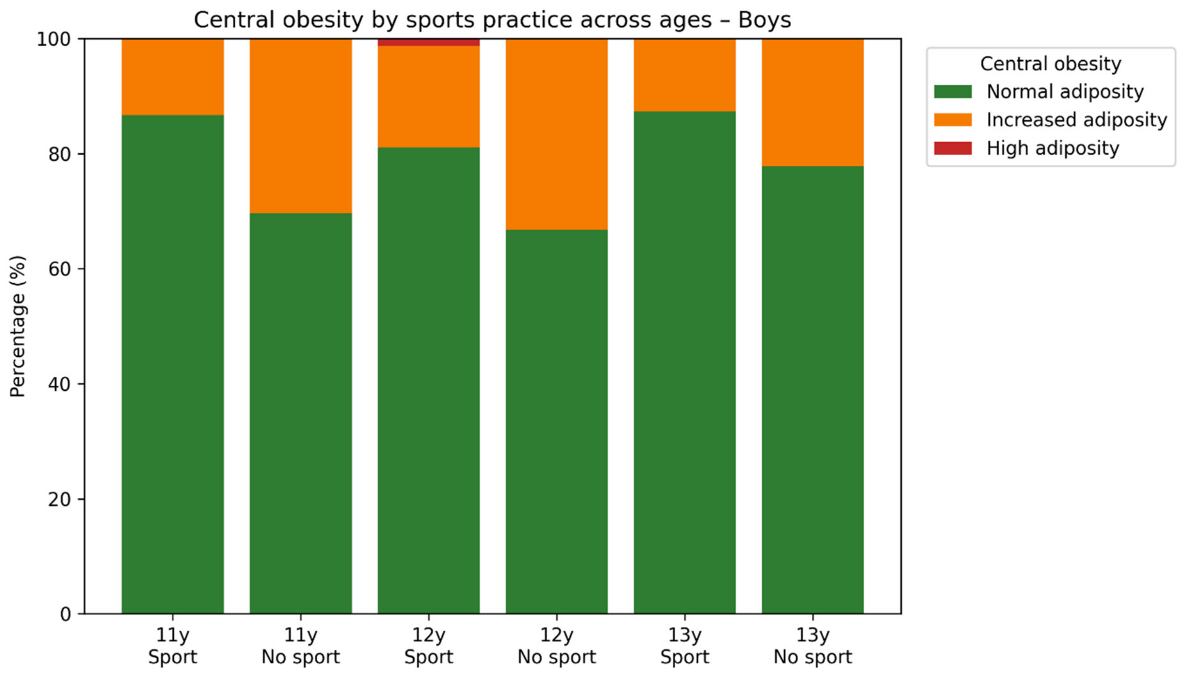

Figure S7. Distribution of central obesity categories according to sports practice (yes/no) across ages (11–13 years) in boys. Data are expressed as percentages.

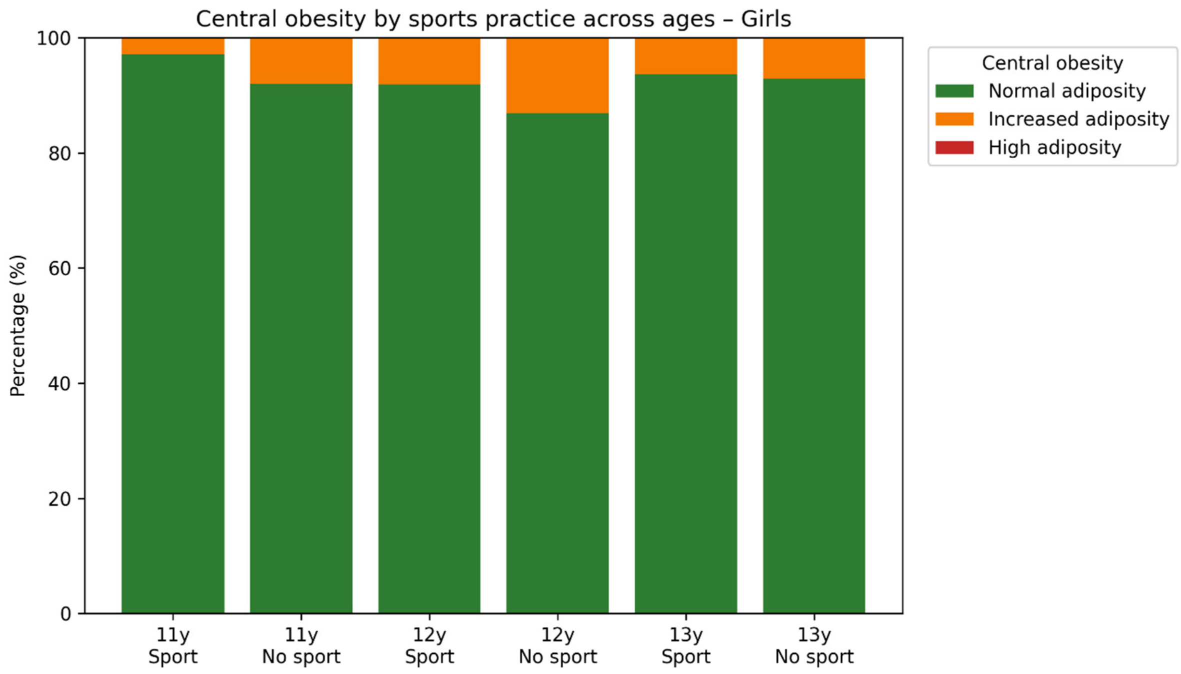

Figure S8. Distribution of central obesity categories according to sports practice (yes/no) across ages (11–13 years) in girls. Data are expressed as percentages.
